# Acute appearance of fatty acids in human plasma – a comparative study between polar-lipid rich oil from the microalgae *Nannochloropsis oculata* and krill oil in healthy young males

**DOI:** 10.1186/1476-511X-12-102

**Published:** 2013-07-15

**Authors:** Michael L Kagan, Annette L West, Christa Zante, Philip C Calder

**Affiliations:** 1 Qualitas Health Ltd., 19 Hartom Street, P.O. Box 45423, Jerusalem 91450, Israel; 2 Human Development and Health Academic Unit, Faculty of Medicine, University of Southampton, IDS Building, MP887 Southampton General Hospital, Tremona Road, Southampton SO16 6YD, UK; 3 MPS Hamburg GmbH, Kieler Strasse 99-105, Hamburg 22769, Germany

**Keywords:** Omega-3, Eicosapentaenoic acid, Docosahexaenoic acid, Algal oil, Krill oil, Polar lipids, Glycolipids, Phospholipids

## Abstract

**Background:**

The long-chain n-3 fatty acids eicosapentaenoic acid (EPA) and docosahexaenoic acid (DHA) have human health benefits. Alternatives to fish as sources of EPA and DHA are needed. Oil from the micro-algae *Nannochloropsis oculata* contains a significant amount of EPA conjugated to phospholipids and glycolipids and no DHA. Krill oil contains EPA and DHA conjugated to phospholipids. We compare the appearance of fatty acids in blood plasma of healthy humans after consuming a high fat meal followed by either algal oil or krill oil.

**Methods:**

Ten healthy males aged 18-45 years consumed a standard high fat (55 g) breakfast followed by either algal oil (providing 1.5 g EPA and no DHA) or krill oil (providing 1.02 g EPA and 0.54 g DHA). All participants consumed both oils in random order and separated by 7 days. Blood samples were collected before the breakfast and at several time points up to 10 hours after taking the oils. Fatty acid concentrations (μg/ml) in plasma were determined by gas chromatography.

**Results:**

Fatty acids derived mainly from the breakfast appeared rapidly in plasma, peaking about 3 hours after consuming the breakfast, and in a pattern that reflected their content in the breakfast. There were time-dependent increases in the concentrations of both EPA and DHA with both algal oil (P < 0.001 for EPA; P = 0.027 for DHA) and krill oil (P < 0.001 for both EPA and DHA). The concentration of EPA was higher with algal oil than with krill oil at several time points. DHA concentration did not differ between oils at any time point. The maximum concentration of EPA was higher with algal oil (P = 0.010) and both the area under the concentration curve (AUC) and the incremental AUC for EPA were greater with algal oil (P = 0.020 and 0.006). There was no difference between oils in the AUC or the incremental AUC for DHA.

**Conclusion:**

This study in healthy young men given a single dose of oil indicates that the polar-lipid rich oil from the algae *Nannochloropis oculata* is a good source of EPA in humans.

## Background

Omega-3 (n-3) fatty acids are a family of polyunsaturated fatty acids (PUFAs) that have a carbon-carbon double bond on the third carbon from the methyl end of the hydrocarbon chain [1]. The two long-chain (LC) n-3 fatty acids of most importance to human health are eicosapentaenoic acid (EPA; 20:5n-3) and docosahexaenoic acid (DHA; 22:6n-3) [1]. Since EPA and DHA are poorly synthesized endogenously in humans [2], they may be essential and hence, must come from external sources. Seafood is a good source of EPA and DHA, and also provides the metabolic intermediate docosapentaenoic acid (DPA; 22:5n-3). Since the original observations in Greenland Inuit of an association between high dietary intake of these marine LC n-3 PUFAs and lowered risk of cardiovascular disease [3,4], effects clearly linked to biological functions of the fatty acids, much interest has been focused on the health benefits of seafood in general and of marine LC n-3 PUFAs in particular [5-10].

EPA and DHA have been shown to lower blood triglycerides [11] and the US Food and Drug Administration has approved two LC n-3 PUFA based prescription drugs for treating hypertriglyceridemia: Lovaza® (GSK), consisting of EPA plus DHA, and Vascepa® (Amarin), consisting of EPA only. Besides lowering blood triglycerides, EPA and DHA also exert beneficial effects in inflammatory conditions [12,13]. Furthermore, there is accumulating evidence that EPA and DHA play an important role in ameliorating psychiatric and psychological disorders in adults [14,15] and in slowing cognitive decline in the elderly [16,17]. As a result of these beneficial effects on human health, particularly the cardio-protective effects, there have been recommendations that individuals should increase their daily intake of LC n-3 fatty acids [18,19]. Consequently, the use of food supplements in the form of oil capsules containing purified or processed fish oil has become popular.

Because of sustainability issues, sources of LC n-3 PUFAs other than fish are likely to be increasingly important. In the last few years, krill oil from *Euphausia superba* has become a recognized commercial source of the LC n-3 fatty acids EPA and DHA [20]. In addition, EPA and DHA have been found to be produced by other organisms including micro-algae [21]. *Crypthecodinium cohnii* dinoflagellate produces DHA only [22], Schizochytrium unicellular protists produce both EPA and DHA [23] and the genetically modified *Yarrowia lipolytica* yeast produces EPA only [24]. In fish oil the LC n-3 fatty acids are primarily conjugated to a triglyceride (TAG) backbone, whereas in krill oil the fatty acids are largely conjugated to phospholipids (PhLs) [25]. PhLs not only play a role in health [26] but there is evidence that LC n-3 PUFAs in PhLs have a higher bioavailability than those in TAGs [27]. These properties suggest that krill oil may have superiority over some other sources of LC n-3 PUFAs [28]. Recently, various species of the genus Nannochloropsis have been found to contain high concentrations of EPA with no DHA [29]. The oil extracted from the micro-algae *Nannochloropsis oculata* comprises up to 8-10% PhLs and 30-40% glycoplipids [30] as polar-lipids with 30-40% EPA as the major fatty acid.

There are a growing number of studies that have shown that EPA and DHA have different biological effects and potencies. Some studies suggest that of these two LC n-3 fatty acids, EPA rather than DHA is primarily responsible for reducing inflammation [31] and depression [32] and that while both EPA and DHA modify blood lipid profiles, DHA seems to have the strongest effects [33]. In a recent meta-analysis of studies in depression [15] it has been suggested that, not only is EPA responsible for therapeutic effects, but that DHA acts to block the activity of EPA. The oil extracted from the micro-algae Nannochloropsis is one of the few sources of EPA without the presence of DHA and thus presents a means of maximizing the efficacy of EPA. An important question that is not well resolved is whether LC n-3 fatty acids (EPA and DHA) are equally available to the human body when provided from algal oil or krill oil in which the fatty acids are present in different forms. Thus, the present study set out to follow the plasma levels of EPA and DHA, as well as of other fatty acids, in young healthy subjects in the period immediately following consumption of algal oil or krill oil with a high fat meal.

## Methods

### Subjects and study design

The study had a cross-over design in which all participants consumed both algal oil and krill oil in random order separated by 7 days. The study was approved by the Ethics Committee of the Ärztekammer Hamburg and the participants provided written informed consent. Inclusion criteria were: male; 18 to 45 years of age, inclusive; body mass index (BMI) ≥ 20 and ≤ 32 kg/m^2^; non-smoker; in general good health on the basis of medical history, and laboratory values, physical examination, vital signs and 12-lead electrocardiogram at screening; willingness to adhere to the study protocol; willingness to refrain from consumption of oily fish more than once a week during the study period. Exclusion criteria were: clinically relevant abnormal laboratory test results at screening; history or presence of clinically important metabolic, endocrine (including type 1 or type 2 diabetes), cardiovascular, hepatic, renal, hematologic, immunologic, gastrointestinal, neurologic, psychiatric or biliary disorders; recent history of (within 12 months of screening) or strong potential for alcohol or substance abuse; con-suming more than one oily fish meal per week; use of any prescription drug within 2 weeks before clinic visit 1 and use of any over-the-counter drug within 1 week before clinic visit 1; use of any anti-inflammatory medication within four weeks of clinic visit 1 or during the study period; use of any lipid-lowering medication within four weeks of clinic visit 1 or during the study period; consumption of fish oil or other oil supplements within 3 weeks before clinic visit 1 or during the study period; known allergy or sensitivity to n-3 fatty acids, fish or seafood; inability to provide written informed consent. Ten subjects were recruited into the study.

The two different oil supplements used were: QH101 polar-lipid rich algal oil from the micro-algae *Nannochloropsis oculata* (Qualitas Health Ltd., Jerusalem, Israel), and a commercially available krill oil supplement (NKO; Neptune Technologies & Bioresources Inc., Quebec, Canada). Both oils were encapsulated in gelatin soft capsules manufactured by Catalent, Eberbach, Germany.

On days 1 and 8 the participants arrived in the morning after overnight fasting. They were provided a high fat breakfast, after which the test oil supplements were immediately administered. The high fat test meal comprised toast with jam or marmalade (to provide the carbohydrate) and a milkshake made from milkshake powder, double cream, oils (safflower, linseed and olive) and water. The total energy content of the meal was 4.3 kJ and it provided 55 g fat, 130 g carbohydrate and 12 g protein. The major fatty acids in the meal were oleic acid (34% of fatty acids), linoleic acid (22.1%), palmitic acid (21.5%), stearic acid (8.4%), and α-linolenic acid (3.7%).

The supplement capsules were swallowed with 200 ml of water to provide ~1.5 g EPA+DHA. For the algal oil this was purely as EPA with no DHA present while for krill oil this was as 1.02 g EPA and 0.54 g DHA. In order to provide this amount of LC n-3 fatty acids 9 capsules of algal oil or 14 capsules of krill oil were administered. Participants had nothing further to drink except water or tea until the last blood sample was collected. A standardized low-fat snack was provided after 6 hours. Blood samples for the analysis of plasma fatty acid concentrations were collected before the breakfast and 0.5, 1, 1.5, 2, 2.5, 3, 4, 5, 6, 8 and 10 hours after taking the supplements. Blood samples were centrifuged at 1500 x g at room temperature for 15 minutes. Plasma was collected and stored at -80°C until shipping to the University of Southampton, Southampton, UK for analysis.

### Quantitative analysis of krill oil and algal oil

Analysis of the fatty acid content of the algal and krill oils was performed by an external laboratory (New Jersey Feed Labs, NJ, USA) according to the AOAC 963.22 standard using a Shimadzu 17A gas chromatograph. The full fatty acid composition of each supplement is presented in Table 1. The phospholipid content of the two oils was elucidated by ^31^P NMR spectroscopy and the glycolipids and cholesterol content by ^1^H NMR spectroscopy. Both methods used a BRUKER Avance III 600 MHz Nuclear Magnetic Resonance Spectrometer performed by Spectral Services, Cologne, Germany. The polar lipid and cholesterol contents of the two supplements are shown in Table 2. Krill oil was rich in phosphatidylcholine and other PhLs, but did not contain glycolipids. Algal oil contained some PhLs and a greater amount of glycolipids.

**Table 1:** Fatty acid composition of study supplements

**Fatty acid**	**Algal oil**	**Krill oil**
Lauric (12:0)	0.33	0.14
Myristic (14:0)	2.48	5.02
Myristoleic (14:1n-5)	-	0.36
Pentadecanoic (15:0)	0.28	0.19
Palmitic (16:0)	9.63	12.05
Palmitoleic (16:1n-7)	11.68	3.31
Heptadecanoic (17:0)	0.11	0.06
Stearic (18:0)	0.14	0.58
Oleic (18:1n-9)	1.06	5.96
Cis-Vaccenic (18:1n-7)	0.35	3.93
Linoleic (18:2n-6)	1.24	1.05
γ-Linolenic (18:3n-6)	0.16	0.08
Dihomo-γ-linolenic (20:3n-6)	0.42	-
Arachidonic (20:4n-6)	5.3	0.37
Eicosapentaenoic (EPA; 20:5n-3)	25.06	13.63
Docosapentaenoic (DPA; 22:5n-3)	-	0.32
Docosahexaenoic (DHA; 22:6n-3)	-	7.17
Others	11.14	5.75
Total	69.38	65.04

Data are % of oil.

**Table 2:** Polar lipid and cholesterol contents of the study supplements

**Lipid**	**Algal oil**	**Krill oil**
Phosphatidylcholine	2.34	26.77
1-Lysophosphatidylcholine	-	0.32
2- Lysophosphatidylcholine	0.73	2.64
Phosphatidylinositol	0.57	-
Phosphatidylethanolamine	-	1.59
Lysophosphatidylethanolamine	-	0.18
N-Acyl-Phosphatidylethanolamine	-	1.52
Phosphatidylglycerol	1.36	-
Other phospholipid	0.94	0.37
Digalactosydiacylglycerol	8.8	-
Monogalactosydiacylglycerol	2.5	-
Total polar lipids	17.25	33.39
Cholesterol	0.05	2.1

Data are % of oil. Neither oil contained detectable amounts of lysophosphatidylinositol, phosphatidylserine, lysophosphatidylserine, sphingolipids, diphosphatidylglycerol, phosphatidic acid or lysophosphatidic acid.

### Fatty acid composition analysis of human plasma

Fatty acids were analyzed in the total lipid fraction of human plasma, essentially using the procedure described in Browning et al. for plasma lipid fractions [34]. Prior to lipid extraction internal standard (di-15:0 phosphatidylcholine; 100 μg) was added to plasma to enable quantification of fatty acids. Thawed plasma (0.6 ml) was mixed vigorously with 5 ml chloroform:methanol (2:1 vol/vol); butylated hydroxytoluene (50 mg/l) was included in the chloroform:methanol as antioxidant. After centrifugation the organic phase that includes the extracted plasma lipid was collected. This was dried down under nitrogen at 40°C and redissolved in 0.5 ml toluene. Fatty acid methyl esters (FAMEs) were formed by incubation of the entire lipid extract with 1 ml methanol containing 2% (vol/vol) H_2_SO_4_ at 50°C for 2 hr. After allowing the tubes to cool, samples were neutralized by addition of 1 ml of a solution of 0.25 M KHCO_3_ and 0.5 M K_2_CO_3_. Then FAMEs were extracted into 1 ml hexane, dried down, redissolved in a small volume (150 μl) of hexane, and separated by gas chromatography. Gas chromatography was performed on a Hewlett Packard 6890 gas chromatograph fitted with a BPX-70 column (30 m × 0.22 mm × 0.25 μm). Inlet temperature was 300°C. Oven temperature was initially 115°C and this was maintained for 2 min post-injection. Then the oven temperature was programmed to increase to 200°C at the rate of 10°C/min, to hold at 200°C for 16 min, and then to increase to 240°C at the rate of 60°C/min and then to hold at 240°C for 2 min. Total run time was 37 min. Helium was used as the carrier gas. FAMEs were detected by a flame ionization detector held at a temperature of 300°C. The instrument was controlled by, and data collected using, HPChemStation (Hewlett Packard). FAMEs were identified by comparison of retention times with those of authentic standards run previously. Absolute concentrations of fatty acids were calculated using the 15:0 internal standard.

### Statistical analyses

Data are presented as mean and SD. The area under the plasma concentration-time curve (both the total area under the concentration curve (AUC) and the incremental area under the concentration curve (IAUC)) from baseline up to the last measurement was calculated by linear up-logarithmic down trapezodial summation. Two-factor analysis of variance with repeated measurements (MANOVA) was applied for analyzing the time x oil effect (the differences in measurements (μg/ml)) over all time points between the oil types. One-way analysis of variance with repeated measurements was applied for analyzing the trend over time (12 time-points) and for testing the hypothesis that the trend is different from 0 within each treatment group. The paired *t*-test was used for testing the statistical significance of the change from time 0 (baseline) to all other time points within each study group and for testing the statistical significance of the difference between krill oil and algal oil. All tests applied were two-tailed, and a p-value of < 0.05 was considered statistically significant. Data were analyzed using the SAS® version 9.1 (SAS Institute, Cary North Carolina, USA) by Medistat, Israel.

## Results

### Subject characteristics

Ten healthy males participated in this study. Their characteristics are shown in Table 3. All participants completed both arms of the trial and all samples were collected. There were no indications of intolerance to either supplement.

**Table 3:** Characteristics of the participants

	
Age (years)	35.3 ± 5.8
Systolic blood pressure (mm-Hg)	123 ± 13
Diastolic blood pressure (mm-Hg)	76.0 ± 6.4
Weight (kg)	82.5 ± 5.4
Body mass index (kg/m^2^)	25.6 ± 1.5

Data are mean ± SD for 10 participants.

### Fasting plasma fatty acids

Fasting concentrations of fatty acids averaged over the two clinic visits are shown in Table 4. The major fatty acids present in fasting plasma were linoleic (27.7% of total plasma fatty acids), oleic (23.4%), and palmitic (23%), followed by stearic (7.1%) and arachidonic (6.6%). Mean fasting concentrations of EPA and DHA were 25.6 and 55.8 μg/ml, respectively, representing 0.75 and 1.63% of total fatty acids, respectively.

**Table 4:** **Fasting concentrations of plasma fatty acids and T**_**max**_**, C**_**max**_**, and Max∆C for each fatty acid following consumption of the test meal with algal oil or krill oil**

		**Algal oil**			**Krill oil**		
**Fatty acid**	**Fasting concentration (μg/ml)**	**Tmax (hr)**	**Cmax (μg/ml)**	**Max∆C (μg/ml)**	**Tmax (hr)**	**Cmax (μg/ml)**	**Max∆C (μg/ml)**
Myristic	37.8 ± 4.7	3.0 ± 1.6	107.7 ± 62.4	66.0 ± 39.3	3.2 ± 1.5	98.7 ± 46.9	65.8 ± 39.8
Palmitic	783.6 ± 97.9	3.1 ± 1.7	1106 ± 448	320.7 ± 222.1	3.2 ± 1.9	1024 ± 332	263.8 ± 142.8
Palmitoleic	67.4 ± 9.1	3.2 ± 1.9	97.2 ± 71.5	27.6 ± 18.8	3.0 ± 2.2	77.1 ± 33.1	24.9 ± 13.8
Heptadecanoic	14.8 ± 2.3	2.4 ± 2.1	27.2 ± 15.1	15.6 ± 12.1	3.7 ± 3.1	32.5 ± 11.5	20.0 ± 10.3
Stearic	242.7 ± 29.8	2.6 ± 1.4	346.9 ± 121.7	103.8 ± 80.7	2.8 ± 1.9	330.6 ± 117.7	90.5 ± 61.8
Oleic	800.1 ± 104.7	3.0 ± 1.8	1203 ± 609	434.2 ± 353.6	3.1 ± 2.0	1141 ± 431	353.3 ± 207.5
Cis-Vaccenic	64.7 ± 8.4	3.0 ± 1.6	83.6 ± 39.5	23.9 ± 18.6	3.5 ± 2.1	89.5 ± 35.7	26.5 ± 16.3
Linoleic	947.4 ± 114.7	3.0 ± 1.8	1152 ± 284	240.1 ±143.4	3.7 ± 2.3	1130 ± 221	177.1 ± 60.8
γ-Linolenic	15.3 ± 1.8	4.0 ± 3.3	20.6 ± 16.9	3.9 ± 2.4	3.4 ± 1.8	15.3 ± 6.1	2.6 ±0.8
α-Linolenic	33.7 ± 4.5	3.3 ± 2.0	78.1 ± 36.5	47.4 ± 23.8	2.9 ± 1.7	77.8 ± 47.4	43.6 ± 27.2
Arachidic	6.1 ± 0.8	3.1 ± 1.9	9.2 ± 4.9	3.4 ± 2.1	3.6 ± 1.8	10.9 ± 3.9	5.0 ±2.4
Dihomo-γ-linolenic	49.7 ± 6.2	5.7 ± 3.8	56.8 ± 13.2	8.9 ± 4.3	6.2 ± 3.3	55.2 ± 12.9	6.4 ± 3.4
Arachidonic	226.7 ± 28.5	6.1 ± 3.8	254.5 ± 56.4	38.1 ± 14.8	6.5 ± 3.7	245.6 ± 67.1	29.2 ± 14.4
EPA	25.6 ± 3.6	6.9 ± 2.2	75.9 ± 24.8	50.1 ± 24.2	7.5 ± 2.0	50.4 ± 14.3*	24.9 ± 6.9**
DPA	19.5 ± 2.6	6.7 ± 3.2	26.1 ± 7.0	7.8 ± 3.8	5.6 ± 2.8	25.0 ± 6.8	5.1 ± 2.4
DHA	55.8 ± 7.1	6.7 ± 2.7	66.7 ± 18.9	13.3 ± 7.9	7.4 ± 2.5	70.1 ± 22.5	14.4 ± 5.5

Data are mean ± SD from 10 participants. * indicates P = 0.010 vs algal oil; ** indicates P = 0.006 vs algal oil.

### Changes in plasma fatty acids

**Figure 1:**
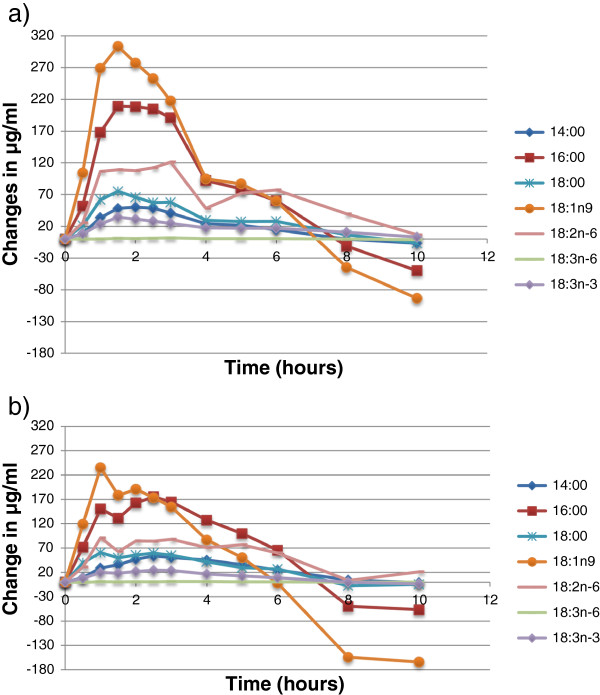
**Changes in the concentrations of 14 to 18 carbon chain length fatty acids when participants consumed a test meal plus a) algal oil or b) krill oil.** Data are mean values from 10 participants. Error bars are omitted for clarity. There were no significant differences between algal oil and krill oil for any fatty acid at any time point.

**Figure 2:**
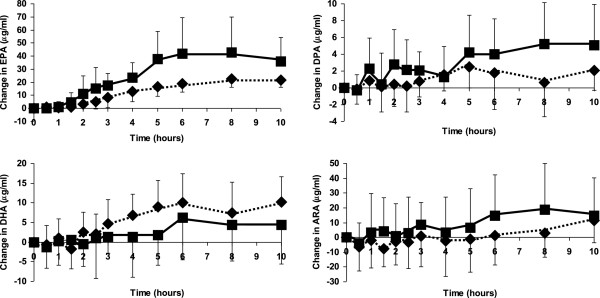
**Changes in the concentrations of 20 and 22 carbon chain length polyunsaturated fatty acids when participants consumed a test meal plus algal oil (closed squares) or krill oil (closed diamonds).** Data are mean values and their standard deviation from 10 participants. Significant differences are as follows: EPA: For both oils 2, 2.5, 3, 4, 5, 6, 8 and 10 hours are significantly different from time zero. Algal oil is significantly greater than krill oil at 5, 6, 8 and 10 hours. DPA: For algal oil 3, 4, 5, 6, 8 and 10 hours are significantly different from time zero. For krill oil 5 and10 hours are significantly different from time zero. DHA: For krill oil 3, 4, 5, 6, 8 and 10 hours are significantly different from time zero.

The changes in concentration of individual fatty acids in plasma are shown in Figure 1, which shows fatty acids of 14 to 18 carbon chain length, and Figure 2, which shows PUFAs of 20 and 22 carbon chain length. Increases in fatty acid concentrations were seen at the first post-meal blood sampling time point (30 minutes). Oleic, palmitic and linoleic acids showed the greatest increases in concentration (Figure 1), which is consistent with these fatty acids being the most abundant in the test meal. There were no differences in the concentration, or in the change in concentration, of any 14 to 18 carbon chain length fatty acids between algal oil and krill oil at any time point (Table 4).

MANOVA revealed significant oil x time interactions for EPA (P = 0.001), DPA (P = 0.032) and DHA (P = 0.049). There were time-dependent increases in the concentration of EPA when the participants consumed either algal oil or krill oil with the test meal (P < 0.001 for both) (Figure 2). There was also an increase in the concentration of DPA with algal oil (P < 0.001) and a trend to an increase in DPA with krill oil (P = 0.083). There were time-dependent increases in the concentration of DHA when the participants consumed either algal oil or krill oil with the test meal (P = 0.027 and P < 0.001, respectively). With both algal and krill oils EPA concentration was higher than at time zero at all time points from 2 hours on. With algal oil DPA concentration was higher than at time zero at all time points from 3 hours on, while with krill oil DPA concentration was higher than at time zero at 5 and 10 hours. With algal oil DHA concentration was not significantly higher than at time zero at any individual time point, while with krill oil DHA concentration was higher than at time zero at all time points from 3 hours on.

EPA concentration was significantly higher with algal oil than with krill oil at 5, 6, 8 and 10 hours (P < 0.05) and tended to be higher at 4 hours (P = 0.094). DPA, DHA and arachidonic acid concentrations did not differ between oils at any time point, although there was a trend for DHA concentration to be higher with krill oil at 5 hours (P = 0.083).

The time at which each fatty acid reached its peak concentration (Tmax) is shown in Table 4. Fatty acids of chain length 18 carbons or less reached their peak concentration about 3 hours after consuming the test meal, while PUFAs of 20 carbons or more reached their peak concentration 6 to 8 hours after consuming the meal. There were no significant differences in Tmax between oils for any fatty acid (Table 4).

Table 4 also shows the maximum concentration (Cmax) for each individual fatty acid. Cmax of EPA was significantly higher with algal oil than with krill oil (P = 0.010). The maximum change in concentration of EPA from its fasting concentration (MaxΔC) was also significantly higher with algal oil than with krill oil (P = 0.006). There were no significant differences in Cmax or MaxΔC between oils for any fatty acid other than EPA (Table 4).

The area under the concentration curve (AUC) and the incremental AUC (IAUC) for each fatty acid are shown in Table 5. The AUC and IAUC for EPA were greater with algal oil compared with krill oil (P = 0.020 and P = 0.006, respectively), but there was no difference for the AUC or IAUC for any other fatty acid including DPA or DHA (Table 5).

**Table 5:** Area under the concentration-time curve (AUC) and incremental AUC for each fatty acid following consumption of the test meal with algal oil or krill oil

	**Algal oil**		**Krill oil**	
**Fatty acid**	**AUC ((μg/ml)/hr)**	**IAUC ((μg/ml)/hr)**	**AUC ((μg/ml)/hr)**	**IAUC ((μg/ml)/hr)**
Myristic	611 ± 400	250 ± 166	580 ± 250	268 ± 169
Palmitic	8670 ± 3161	1398 ± 931	8394 ± 2432	1170 ± 526
Palmitoleic	753 ± 596	128 ± 93	697 ± 257	105 ± 51
Heptadecanoic	144 ± 62	74 ± 88	165 ± 47	62 ± 30
Stearic	2727 ± 697	419 ± 299	2684 ± 758	365 ± 200
Oleic	8580 ± 3739	1851 ± 1289	8502 ± 2711	1614 ± 968
Cis-Vaccenic	648 ± 251	107 ± 69	689 ± 233	107 ± 62
Linoleic	10025 ± 2027	1102 ± 737	10074 ± 1997	800 ± 337
γ-Linolenic	180 ± 153	22 ± 17	136 ± 59	11 ± 4
α-Linolenic	473 ± 218	198 ± 120	469 ± 216	191 ± 126
Arachidic	69 ± 41	16 ± 13	75 ± 28	17 ± 10
Dihomo-γ-linolenic	518 ± 137	44 ± 31	513 ± 122	31 ± 15
Arachidonic	2342 ± 550	214 ± 107	2290 ± 646	134 ± 81
EPA	533 ± 157	277 ± 135	391 ± 120*	137 ± 39**
DPA	220 ± 58	39 ± 26	214 ± 58	25 ± 12
DHA	582 ± 164	65 ± 45	621 ± 205	70 ± 39

Data are mean ± SD from 10 participants. * indicates P = 0.020 vs algal oil; ** indicates P = 0.006 vs algal oil.

**Figure 3:**
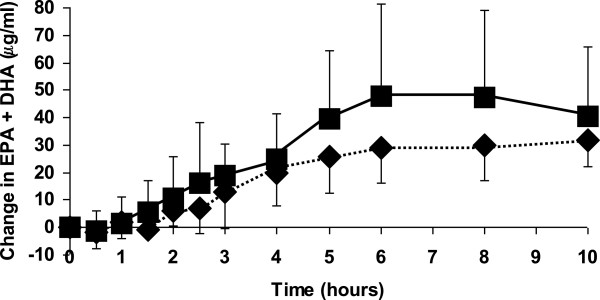
**Changes in the concentrations of EPA+DHA when participants consumed a test meal plus algal oil (closed squares) or krill oil (closed diamonds).** Data are mean values and their standard deviation from 10 participants. Significant differences are as follows: For algal oil 2.5, 3, 4, 5, 6, 8 and 10 hours are significantly different from time zero. For krill oil 2, 2.5, 3, 4, 5, 6, 8 and 10 hours are significantly different from time zero. There are no differences between oils at any time point.

Figure 3 shows the change in the concentration of EPA plus DHA in plasma. Cmax of EPA plus DHA tended to be higher with algal oil (P = 0.098) and MaxΔC was significantly higher with algal oil (P = 0.035). IAUC for EPA plus DHA also tended to be higher with algal oil (P = 0.076). There was no difference between oils at any time point.

## Discussion

Here we report the changes in fatty acid concentrations in plasma over 10 hours following consumption of a high fat breakfast plus supplements providing LC n-3 PUFAs. Some fatty acids in plasma are in the “free” (i.e. non-esterified) form but the bulk are esterified to produce more complex lipids mainly TAGs, PhLs, and cholesteryl esters. In turn, the complex lipids are components of lipoproteins whose role is to transport lipids, especially the fatty acid constituents, between tissues. In lipoproteins, the non-polar lipids (TAGs, cholesteryl esters) form a core which is surrounded by a PhL monolayer. The TAGs, cholesteryl esters and PhLs have characteristic fatty acid compositions but both the complex lipid content and the fatty acid composition of lipoproteins changes as they are metabolised. After eating, the bulk of fatty acids derived from the meal appear in the circulation as components of TAGs within chylomicrons. In the current study fatty acids of 14 to 18 carbons derived from the meal appeared quickly in plasma and reached their maximal concentration around 3 hours after consuming the breakfast. This is entirely consistent with the time course of the change in concentration of total TAGs [35-37] and of chylomicrons [35,37]. The changes in the concentrations of 14 to 18 carbon fatty acids in plasma are in accordance with the relative amounts of those fatty acids in the breakfast. This agrees with the data presented by Burdge et al. [36] and fits with current concepts on the handling of dietary fat [38]. Most of these fatty acids had returned to their starting concentrations by 8 hours following the breakfast.

In contrast to 14 to 18 carbon fatty acids, 20 and 22 carbon PUFAs demonstrated a slower rise in concentration in plasma and reached their maximum concentrations 6 to 8 hours after consuming the breakfast. Again this pattern of appearance is in agreement with the literature. Burdge et al. [39] reported appearance of EPA and DHA in plasma TAGs after subjects consumed a high fat breakfast containing EPA and DHA and saw that the maximum concentrations were not reached until 4 hours after completing the meal and then were sustained for a further two hours. They did not make measurements beyond that time point. In the current study EPA and DHA concentrations were maintained at their maximum until 10 hours after the meal was consumed. Heath et al. [35] reported EPA and DHA in chylomicron-like particles, chylomicron remnants, very low density lipoproteins (VLDLs) and non-esterified fatty acids (NEFAs) over 6 hours following their consumption. The appearance of EPA and DHA in chylomicron-like particles was maximal at 3 and 4 hours. They then tracked into chylomicron remnants peaking in concentration at 4.5 hours. Finally, they appeared in VLDLs peaking in concentration at 5-6 hours. EPA concentration was greater than DHA in the chylomicron-like particles and the chylomicron remnants, reflecting the higher content of EPA than DHA in the test meal [35]. However, DHA concentration was greater than that of EPA in VLDLs [35], indicating different pathways of metabolism and handling of EPA and DHA over the 6 hours following their consumption in a meal. Heath et al. [35] also reported appearance of EPA and DHA, with the concentration of DHA being greater, in NEFAs in the late phase of their study. The sequential movement of EPA and DHA through the various lipoproteins and the relative enrichment of VLDLs in DHA were confirmed in a second study by Heath et al. [37]. The later appearance of EPA and DHA in plasma compared with shorter chain fatty acids observed in the current study is consistent with the findings of Burdge et al. and Heath et al. in relation to the late appearance of EPA and DHA in plasma TAGs and TAG-rich lipoproteins. It seems likely that in the current study the EPA and DHA appearing in the plasma over the first hours is within chylomicrons, as seen by Heath et al. [35], but that later on these fatty acids are within remnant particles, VLDLs and NEFAs.

Plasma EPA concentration increased with both algal and krill oils, but was higher with algal oil reflecting its higher EPA content. The mean increase in plasma EPA with algal oil was 100% higher than with krill oil. Participants consumed 1.5 g EPA from algal oil and 1 g EPA from krill oil. This suggests that on a gram-per-gram basis algal oil may be a more effective source of EPA than krill oil. It is possible that the presence of DHA in krill oil limits the incorporation of EPA into plasma lipids. Another possibility is that n-3 PUFAs within glycolipids, as found in algal oil but not krill oil, are an effective system for delivering EPA to humans. The apparently higher incorporation of EPA with algal compared with krill oil is particularly interesting because a recent study reported better incorporation of EPA into plasma over 72 hours if participants consumed krill oil compared with if they consumed TAGs, both providing the same amounts of EPA and DHA [27]. Taking this finding together with that of the current study suggests that an EPA-rich algal oil may be a very effective way of enhancing EPA status in humans. This will need to be studied in longer term intervention studies that assess other functional pools such as leukocytes and red cells [34] and link the fatty acid changes observed to physiological effects of relevance to human health [1].

Plasma DHA concentration clearly increased with krill oil as shown by the significant relationship between time and DHA concentration and by the significant differences in DHA concentration between time zero and all time points from 3 hours on when the participants consumed krill oil. Nevertheless, plasma DHA concentration did not differ between algal and krill oils at any time point, even though krill oil contains DHA and the algal oil does not. This is because there was a small increase in concentration of DHA with algal oil seen as a significant relationship between time and DHA concentration. It is not clear why this might be, although the effect is sufficiently small that there was no difference in DHA concentration between time zero and any subsequent time point when participants consumed algal oil. It is generally considered that there is poor conversion of EPA to DPA and especially to DHA [2,40]. It is possible that enterocytes may be able to carry out this metabolic transformation, but the rather late appearance of DPA and DHA with algal oil suggests that enterocyte biosynthesis of these two fatty acids is not likely to be involved. There is more likely to be a metabolic explanation for these observations. It is possible that DHA, and perhaps also DPA, become preferentially concentrated in VLDLs late in the post-prandial period, as suggested by the observations of Heath et al. [35]. Thus, the appearance of increasing concentrations of DHA in plasma beyond 5 to 6 hours with algal oil may reflect continued accumulation of DHA in lipoproteins synthesised in the liver (VLDLs) using DHA from remnant lipoproteins and NEFAs while at the same time limiting, or perhaps preventing, DHA oxidation.

## Conclusions

Consuming a polar-lipid rich oil from the microalgae *Nannochloropsis oculata* or krill oil results in significant appearance of EPA in human plasma. There is also appearance of DPA and DHA. Algal oil results in a greater concentration of EPA in plasma than krill oil, even taking into account the different EPA contents of the two oils. This difference may relate to the different chemical constituents of the two oils namely the presence of glycolipids. This study demonstrates that polar-lipid rich oil from *Nannochloropsis oculata* is an effective source of EPA in humans.

## Abbreviations

AUC: Area under the concentration-time curve; Cmax: Maximum concentration; DHA: Docosahexaenoic acid; DPA: Docosapentaenoic acid; EPA: Eicosapentaenoic acid; IAUC: Incremental area under the concentration-time curve; LC: Long-chain; Max∆C: Maximum change in concentration; NEFA: Non-esterfied fatty acid; PhL: Phospholipid; PUFA: Polyunsaturated fatty acid; TAG: Triglyceride; Tmax: Time at which maximal concentration is reached; VLDL: Very low density lipoprotein.

## Competing interests

MLK is an employee of Qualitas Health. CZ is an employeee of MPS Hamburg, a contract research organisation. PCC serves on Scientific Advisory Boards of Danone Research Center in Specialised Nutrition, Pronova Biopharma, Aker Biomarine and Smartfish; acts as a consultant to Mead Johnson Nutritionals, Abbott Laboratories, Vifor Pharma, Amarin and Qualitas Health; has received speaking fees from Fresenius-Kabi, BBraun Melsungen, DSM, Nestle, Unilever and Abbott Laboratories; and currently has research funding from Vifor Pharma. ALW has no competing interests to declare.

## Authors’ contributions

MLK and PCC designed the study. CZ obtained ethical approval, recruited participants, conducted the intervention, and collected and processed the blood samples. ALW performed the fatty acid composition analyses under the supervision of PCC. MLK analysed the data and drafted the paper. All authors read and approved the paper.
